# Invasive nontyphoidal *Salmonella* infection in a patient with Roux-en-Y gastric bypass and gastrostomy tube: a case report

**DOI:** 10.1186/s13256-021-03003-0

**Published:** 2021-08-05

**Authors:** Luxi Chen, Temitayo Olojo, Bader Aldeen Alhafez

**Affiliations:** 1 Medical Scientist Training Program, The Ohio State University College of Medicine, Columbus, USA; 2 Department of Internal Medicine, Division of Hospital Medicine, The Ohio State University Wexner Medical Center, M112 Starling Loving Hall, 320 West 10th Avenue, Columbus, OH 43210 USA

**Keywords:** Nontyphoidal *Salmonella*, Bacteremia, Bariatric surgery

## Abstract

**Background:**

Infections with nontyphoidal *Salmonella* most commonly cause a self-limited gastroenteritis in humans and are the leading source of foodborne illness. In the USA, the incidence of culture-confirmed invasive *Salmonella* is extremely rare. Here, we present a unique case of enteric nontyphoidal *Salmonella enteritidis* infection that progressed to an invasive bacteremia in a patient with Roux-en-Y gastric bypass and gastrostomy feeding tube.

**Case presentation:**

A 58-year-old Caucasian woman with a past medical history of Roux-en-Y gastric bypass with revision and recent gastrostomy feeding tube insertion was admitted with fever and diarrhea. During her inpatient stay, two sets of blood cultures grew *Salmonella enteritidis* and she was promptly treated with intravenous antibiotics. Her hospital course was also complicated by acute kidney injury. The patient recovered after treatment with a third-generation cephalosporin and supportive treatment.

**Conclusion:**

We present an unusual case of nontyphoidal *Salmonella enteritidis* bacteremia complicated by acute kidney injury in a middle-aged woman with a previous history of Roux-en-Y gastric bypass and gastrostomy feeding tube insertion. Further investigation is needed to identify whether gastric surgeries or procedures are independent risk factors for invasive nontyphoidal salmonellosis.

## Introduction

Salmonellae are a genus of motile, facultative intracellular Gram-negative bacilli that belong to the Enterobacteriaceae family [1]. As one of the most common causes of foodborne illness, *Salmonella* infection is a major global public health concern. *Salmonella* is transmitted to humans via the fecal–oral route, often through contaminated foods of animal origin such as poultry, beef, fish, milk, and eggs. *Salmonella* is divided into two subspecies, nontyphoidal and typhoidal, that are genetically similar but cause distinct disease states and immune responses in humans. Nontyphoidal serovars typically cause a self-limiting gastrointestinal (GI) disease, while typhoidal serovars cause enteric or typhoid fever, an invasive, systemic, and life-threatening disease that results in more than 200,000 deaths annually worldwide [2].

Nontyphoidal *Salmonella* are a leading cause of bacterial diarrhea. Although the majority of nontyphoidal *Salmonella* infections result in a self-limiting, mild-to-moderate gastroenteritis with low fatality, approximately 1–4% of enteric infections lead to bacteremia [3]. The greatest burden of nontyphoidal *Salmonella* bacteremia is present in Sub-Saharan Africa, accounting for 79% of the total number of cases globally [4]. Causes of serious GI infections may include overuse of antimicrobials, radiation, and immunocompromised states such as cancer and obesity [5–7]. A high incidence of invasive disease occurs in children younger than 5 years of age, the elderly, and immunocompromised individuals, particularly those with human immunodeficiency virus (HIV) infection. In the USA, the mean population incidence of culture-confirmed invasive *Salmonella* is rare, roughly 0.9 cases per 100,000 people annually, mostly occurring in infants [8]. Despite its rarity, invasive *Salmonella* bacteremia typically requires hospitalization as it can be severe and life-threatening [9].

Here, we present an unusual case of nontyphoidal *Salmonella* infection that progressed from enteric to invasive bacteremia in an adult patient with a previous history of Roux-en-Y gastric bypass. Our patient lacked the classic predisposing risk factors for this invasive infection, namely extremes of age, chronic liver disease, and immunosuppressing conditions [10]. We discuss less common risk factors that could alter the GI tract’s microenvironment, such as gastric surgeries and chronic proton pump inhibitor (PPI) use, as they could contribute to the progression from enteric to systemic *Salmonella* bacteremia. Therefore, these risk factors should be considered in the evaluation of the appropriate patient population.

## Case presentation

A 58-year-old Caucasian female presented to her local emergency department with fever and diarrhea. The patient developed nonbloody, large-volume diarrhea after eating take-out 11 days ago, with four to five bowel movements per day. During this time, she also experienced fevers, chills, nausea, vomiting, general malaise, and weakness. In addition, she observed leaking and drainage around her G-tube site with associated erythema and intense abdominal pain. The patient denied sick contacts or recent travel.

Her past medical history includes obesity, orthostatic hypotension, hyperlipidemia, chronic kidney disease, nephrolithiasis, gastrojejunal ulcer, Kawasaki’s disease, subdural hemorrhage, epilepsy, chronic pain syndrome, and anxiety. The patient’s past surgical history includes burr-hole craniotomy, lithotripsy, cholecystectomy, Roux-en-Y gastric bypass complicated by chronic refractory marginal ulceration and small bowel obstruction requiring two resections, ventral hernia repair, and gastrostomy tube insertion.

The patient was pregnant six times and had six miscarriages (G6P0060). She lives with her husband and adopted son with good social support. She is currently unemployed. The patient denies ever smoking and does not drink alcohol. Familial history consists of diabetes, heart disease, and nephrolithiasis in the patient’s mother. Her father had glaucoma, nephrolithiasis, and prostate cancer.

Medications before admission included pantoprazole 40 mg twice a day, sucralfate 1 g twice a day, ondansetron 4 mg twice a day as needed for nausea, midodrine 10 mg three times a day, fludrocortisone 0.1 mg twice a day, atorvastatin 40 mg daily, levetiracetam 1500 twice a day, methadone 10 mg every 8 hours, and lorazepam 0.5 mg daily as needed for anxiety. She was also on daily vitamin A, D3, and B-complex supplements and a monthly cyanocobalamin 1000 μg intramuscular injection, in addition to a nutritional supplement Isosource 1.5 Cal that is administered through the G-tube once every night.

Due to her complex past surgical history, the patient was sent from her local emergency department to our hospital. At presentation, the patient was febrile to 101 ℉. Blood pressure was 110/72 mmHg, and heart rate was 84 beats per minute. Further examination revealed diffuse abdominal tenderness with erythema and purulent drainage around the G-tube site on the left upper quadrant. The general examination showed a thin patient who is alert and oriented. Mucous membranes were moist. Lungs were clear to auscultation as she was breathing comfortably on room air. Heart sounds were regular without murmurs. Extremities were warm with intact pulses bilaterally and no edema. However, she had moderate muscle loss for her age. Skin was intact except for the previously mentioned erythema around the G-tube. Neurological examination revealed normal speech with comprehension, intact cranial nerves II–VII, XI, and XII, and 5/5 motor strength in all four extremities. Sensation was intact to light touch and pinprick throughout. Romberg test was negative. Gait was slowed owing to weakness but showed normal balance.

**Fig. 1:**
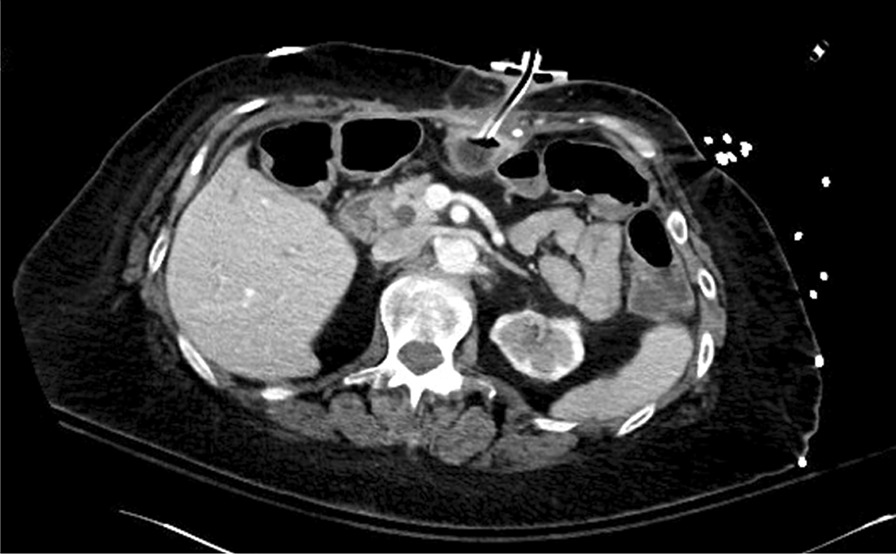
Computed tomography scan of the abdomen showing Roux-en-Y gastric bypass anatomy with patent gastrojejunostomy. A percutaneous gastrostomy tube is present in the excluded portion of the stomach in an appropriate position. Some skin thickening and subcutaneous fat stranding are seen along the insertion site

Serum chemistry showed sodium of 136 mmol/L, potassium of 2.9 mmol/L, chloride of 111 mmol/L, bicarbonate of 20 mmol/L, magnesium 1.2 mg/dL, calcium 6.1 mg/dL, and serum creatinine 1.0 mg/dL. Liver function tests showed low albumin of 2.5 g/dL, total bilirubin of 0.3 mg/dL, alkaline phosphatase of 86 U/L, alanine aminotransferase (ALT) of 13 U/L and aspartate aminotransferase (AST) of 21 U/L. Complete blood count showed a white blood cell count of 3.97 × 10^9^ /L, hemoglobin of 12.6 g/dL, hematocrit of 39.6%, and platelet count of 239 × 10^9^ /L. Urinalysis showed normal appearance and color, specific gravity of 1.020, without detecting red blood cells (RBC), white blood cells (WBC), ketones, glucose, leukocytes esterase, or nitrites. A stool sample was sent for *Clostridium difficile* and a molecular enteric panel but was pending at that time. Two out of two blood cultures that were collected at her local emergency department were positive for Gram-negative rods. An abdominal radiograph confirmed G-tube location overlying the stomach and a computed tomography (CT) abdomen/pelvis with contrast showed nonspecific skin thickening and subcutaneous fat stranding along the G-tube insertion site (Fig. 1). Due to a prior history of penicillin and sulfa anaphylactic reactions, the patient was empirically treated with intravenous ciprofloxacin 400 mg every 8 hours for bacteremia, and oral clindamycin 450 mg every 8 hours for the cellulitis surrounding her G-tube site, in addition to intravenous fluid resuscitation with normal saline at 75 mL/hour and electrolyte repletion with oral potassium chloride 20 mEq for four total doses, and intravenous magnesium sulfate 4 g once.

A fluoroscopy G-tube study revealed no contrast leak or evidence of obstruction. On day 1 of hospitalization, the molecular enteric stool panel was positive for the *Salmonella* species. On day 4, two out of two blood cultures grew *Salmonella enteritidis* with susceptibilities to ampicillin, ceftriaxone, and trimethoprim/sulfamethoxazole. The patient was then transitioned from intravenous ciprofloxacin to a 14-day course of intravenous ceftriaxone 2 g every 24 hours. We resumed tube feeds through the G-tube on day 5 after a successful clamping trial. On day 6, the patient was somnolent and difficult to arouse, with poor skin turgor and dry mucous membranes. Vital signs remained within normal limits; T max of 98.6 ℉, blood pressure of 98/65 mmHg, and heart rate of 95 beats per minute. Laboratory results were significant for a serum creatinine level of 5.25 mg/dL and non-anion gap metabolic acidosis with sodium of 131 mmol/L, chloride of 105 mmol/L, and serum bicarbonate of 18 mmol/L. These clinical and laboratory findings of dehydration suggested the development of prerenal acute kidney injury (AKI) in the setting of prolonged GI volume loss. The patient was resuscitated with half normal saline containing 75 mEq sodium bicarbonate additive at 125 mL/hour. On day 8, the patient’s mental status improved but her G-tube began to leak, prompting clamping of the G-tube. The patient was encouraged to increase oral intake. Intravenous fluids were discontinued on day 9, as she was able to tolerate oral foods and liquids well. No further leakage was seen around her G-tube site. On day 13, the patient was discharged home with a peripherally inserted central line to finish a 14-day course of intravenous ceftriaxone (2 g every 24 hours).

Following completion of a 14-day course of intravenous ceftriaxone, the patient made a full recovery with resolution of her diarrhea and abdominal pain. Tube feeds were resumed without further issues. At a 4-month follow-up visit with her bariatric surgeon, the patient reports that she is doing well and happy with her progress. Electrolytes and serum creatinine were all within normal limits. The patient was readmitted 6 months following her initial hospitalization for abdominal pain and hematemesis due to a large marginal ulcer. She improved with conservative measures including bowel rest (nothing per os) and maintenance intravenous fluids, and was discharged 2 days later. At the time of discharge, her hemoglobin remained stable at 11.2 g/dL, and she was able to tolerate an oral diet. She continues to follow up with her bariatric surgeon in clinic with no recurrence of fever or diarrhea.

## Discussion

In summary, we present a unique case of nontyphoidal *Salmonella enteritidis* bacteremia complicated by acute kidney injury in a middle-aged woman with a previous history of Roux-en-Y gastric bypass and G-tube insertion. The patient initially developed *Salmonella* gastroenteritis through a fecal–oral route, which progressed to invasive bacteremia likely secondary to alterations of the GI tract and malnutrition. She was successfully treated with a 14-day course of intravenous ceftriaxone and fluid resuscitation.

Although the patient lacked the classic predisposing risk factors for invasive nontyphoidal *Salmonella* infection, namely extremes of age, chronic liver disease, malaria, and immunosuppressing conditions [10], her past medical history of Roux-en-Y gastric bypass with chronic PPI use likely altered the microenvironment of her GI tract and contributed to the progression from enteric to systemic *Salmonella* bacteremia. Patients who undergo gastric bypass surgery are often prescribed prophylactic proton pump inhibitors (PPIs) post-surgery to reduce marginal ulceration [11]. Chronic PPI use decreases gastric acid secretions and increases the risk of infections that are transmitted via the fecal–oral route [12]. When long-term PPI administration suppresses gastric acid secretion in the GI tract, there is a higher susceptibility to infection and proliferation of acid-labile bacteria such as *Salmonella*. Furthermore, recent studies have revealed that, within a month of PPI initiation, there is a significant decrease in the diversity of normal gut flora [13]. These alterations in the intestinal microbiome can result in the proliferation of pathogenic bacteria, leading to activation of immune cells and cytokine mediators that trigger intestinal inflammation. In the case of our patient, her chronic PPI use since undergoing bariatric surgery in 2004 likely predisposed her to GI tract infections and led to the progression of an enteric *Salmonella* infection to invasive bacteremia.

Interestingly, Siddiqui *et al.* recently reported a case in which a patient developed chronic *Salmonella* infection status post Roux-en-Y gastric bypass surgery that was subsequently treated successfully with laparoscopic cholecystectomy [14]. Of note, the patient in this case report had positive stool and urine cultures for *Salmonella* but negative blood cultures. Similarly, Shogbesan *et al.* described a case of *Shigella sonnei* bacteremia in an otherwise healthy patient with a prior history of Roux-en-Y gastric bypass [15]. The gut itself has been shown to serve as a clinically significant portal of entry for bacterial infection and bacteremia, particularly in the setting of nutritional depletion combined with exposure to endotoxins [16]. Future investigations are warranted to establish whether gastric surgeries and/or procedures independently predispose to the development of *Salmonella* bacteremia in the absence of other risk factors.

Several studies have shown that severe, chronic malnutrition plays an important role in the development of invasive *Salmonella* disease. In Sub-Saharan Africa, a distinct genotype of nontyphoidal *Salmonella enterica*, ST313, has adapted in malnourished patient populations to cause invasive disease [17]. In Kenya, roughly one-third of all pediatric cases of nontyphoidal *Salmonella* bacteremia occur in children with severe malnutrition [18]. Malnutrition has been shown to cause immunodeficiency and altered GI tract structure and function, reducing the ability of the immune system to protect against infections [19]. Indeed, by the time our patient presented to the hospital, she already experienced 11 days of severe diarrhea and poor oral intake due to nausea and vomiting. In addition, she was unable to supplement her diet through her G-tube owing to infection. These factors, along with laboratory values showing hypocalcemia, hypokalemia, and hypomagnesemia, suggest that the patient was suffering from malnutrition that possibly triggered the development of *Salmonella* bacteremia.

During the patient’s hospital stay, she also experienced acute kidney injury with rising serum creatinine levels. Acute renal dysfunction has been described in previous studies in the setting of severe *Salmonella* infection and is commonly attributed to dehydration due to GI losses, shock, rhabdomyolysis, or intrinsic renal disease such as acute tubular necrosis [20]. In fact, the incidence of renal impairment accompanying *Salmonella* gastroenteritis is fivefold higher compared with that caused by other gastrointestinal pathogens. Overall, the prognosis of acute kidney injury as a result of *Salmonella* infection appears to have a benign course. A majority of currently reported cases resolved successfully with aggressive fluid replacement and appropriate antibiotic treatment without the need for hemodialysis, as with our patient [20–22]. Thus, early recognition and prompt treatment of acute kidney injury are especially important in the context of *Salmonella* gastroenteritis and/or bacteremia.

*Salmonella enteritidis* is the third most common serotype to cause culture-confirmed invasive *Salmonella* infections in the USA [8]. Nontyphoidal *Salmonella* bacteremia is treated empirically with either intravenous fluoroquinolones or third-generation cephalosporins and tailored once susceptibility data become available. The duration of antimicrobial therapy varies, with longer courses warranted for patients with significant immunosuppression [23]. While our patient was successfully treated with a 2-week course of intravenous ceftriaxone, reduced susceptibility and multidrug resistance to fluoroquinolones and third-generation cephalosporins remain a major public health issue worldwide. In Sub-Saharan Africa, multidrug resistance was observed in 75% of nontyphoidal *Salmonella* isolates [24]. In the USA, the number of ceftriaxone-resistant bloodstream isolates doubled from 2.5% between 1996 and 2007 to 5% between 2003 and 2013 [25]. Thus, sound judgment in antimicrobial use in both humans and food-producing animals is paramount to limit the spread of antibiotic resistance and emergence of more virulent strains of *Salmonella*.

## Conclusion

In conclusion, we present an unusual case of nontyphoidal *Salmonella enteritidis* bacteremia complicated by acute kidney injury in an adult patient with a prior history of Roux-en-Y gastric bypass and G-tube insertion that was successfully treated with a 2-week course of intravenous antibiotics and extensive fluid resuscitation. The cause of the patient’s *Salmonella* bacteremia was most likely multifactorial, due to an altered microbiome status post bariatric surgery, chronic PPI use, and malnutrition secondary to large volume diarrhea and poor oral intake. In patients with a Roux-en-Y gastric bypass who develop *Salmonella* gastroenteritis, there should be a high index of suspicion for possible systemic spread as early recognition is important to initiate appropriate management and prevent complications. Further investigation is necessary to identify whether gastric surgeries or procedures are independent risk factors for invasive nontyphoidal salmonellosis.

## Data Availability

Not applicable.
